# Rationalizing Heterogeneity in *Staphylococcus aureus* Bacteremia: Current Progress and Future Goals

**DOI:** 10.1093/infdis/jiag327

**Published:** 2026-06-30

**Authors:** Clark D Russell, Sofía De La Villa, Maaike C Swets, Annette C Westgeest, Giulia Zumbo, Patricia Muñoz, David H Dockrell, Vance G Fowler, Belén Gutiérrez-Gutiérrez

**Affiliations:** Centre for Inflammation Research, Institute for Regeneration and Repair, The University of Edinburgh, Edinburgh, United Kingdom; Baillie Gifford Pandemic Science Hub, Institute for Regeneration and Repair, The University of Edinburgh, Edinburgh, United Kingdom; Clinical Infection Research Group Edinburgh, Western General Hospital, Edinburgh, United Kingdom; Clinical Microbiology and Infectious Diseases Department, Hospital General Universitario Gregorio Marañón, Madrid/Instituto de Investigación Sanitaria Gregorio Marañón (IiSGM), Madrid, Spain; Biomedical Research Networking Centre for Infectious Diseases (CIBERINFEC), Instituto de Salud Carlos III, Madrid, Spain; Department of Internal Medicine, Haaglanden Medical Center, The Hague, The Netherlands; Leiden University Center for Infectious Diseases, Leiden University Medical Center, Leiden University, Leiden, The Netherlands; Division of Infectious Diseases and Microbiology, Virgen Macarena University Hospital, Seville, Spain; Department of Medicine, University of Seville, Seville, Spain; Institute of Biomedicine of Seville (IBiS)/CSIC, Seville, Spain; Clinical Microbiology and Infectious Diseases Department, Hospital General Universitario Gregorio Marañón, Madrid/Instituto de Investigación Sanitaria Gregorio Marañón (IiSGM), Madrid, Spain; Centre for Inflammation Research, Institute for Regeneration and Repair, The University of Edinburgh, Edinburgh, United Kingdom; Clinical Infection Research Group Edinburgh, Western General Hospital, Edinburgh, United Kingdom; Division of Infectious Diseases and International Health, Department of Medicine, Duke University School of Medicine, Durham, North Carolina, USA; Biomedical Research Networking Centre for Infectious Diseases (CIBERINFEC), Instituto de Salud Carlos III, Madrid, Spain; Division of Infectious Diseases and Microbiology, Virgen Macarena University Hospital, Seville, Spain; Department of Medicine, University of Seville, Seville, Spain; Institute of Biomedicine of Seville (IBiS)/CSIC, Seville, Spain

**Keywords:** *Staphylococcus aureus* bacteremia, subphenotypes, endotypes, patient stratification, personalized medicine

## Abstract

Clinical and biological heterogeneity in *Staphylococcus aureus* bacteremia (SAB) continue to pose significant challenges for the clinical management of individual patients and for research efforts. We contend that investigating SAB as a single disease is impeding the identification of beneficial therapeutic approaches. Recently, three data-driven approaches for patient stratification in SAB have been reported, using routinely available data to identify clinically distinct subphenotypes with differences in mortality, microbiologic outcomes, and response to adjunctive antimicrobial therapies (the Swets et al, FEN-AUREUS, and MRSA-GEIRAS-SEIMC studies). In this review, we compare the methodologies and findings from these studies, discuss their limitations, and identify goals for future research in this space. We expect that transformative advances in SAB management will only be achieved by addressing clinical and biological heterogeneity, enabling progress from observable clinical subphenotypes to mechanism-based endotypes defined by treatable traits.

## Teaser

We review current data-driven approaches for patient stratification in SAB, discuss their limitations, and identify goals for future research.

## INTRODUCTION: CLINICAL HETEROGENEITY IN *STAPHYLOCOCCUS AUREUS* BACTEREMIA

**Figure 1.**
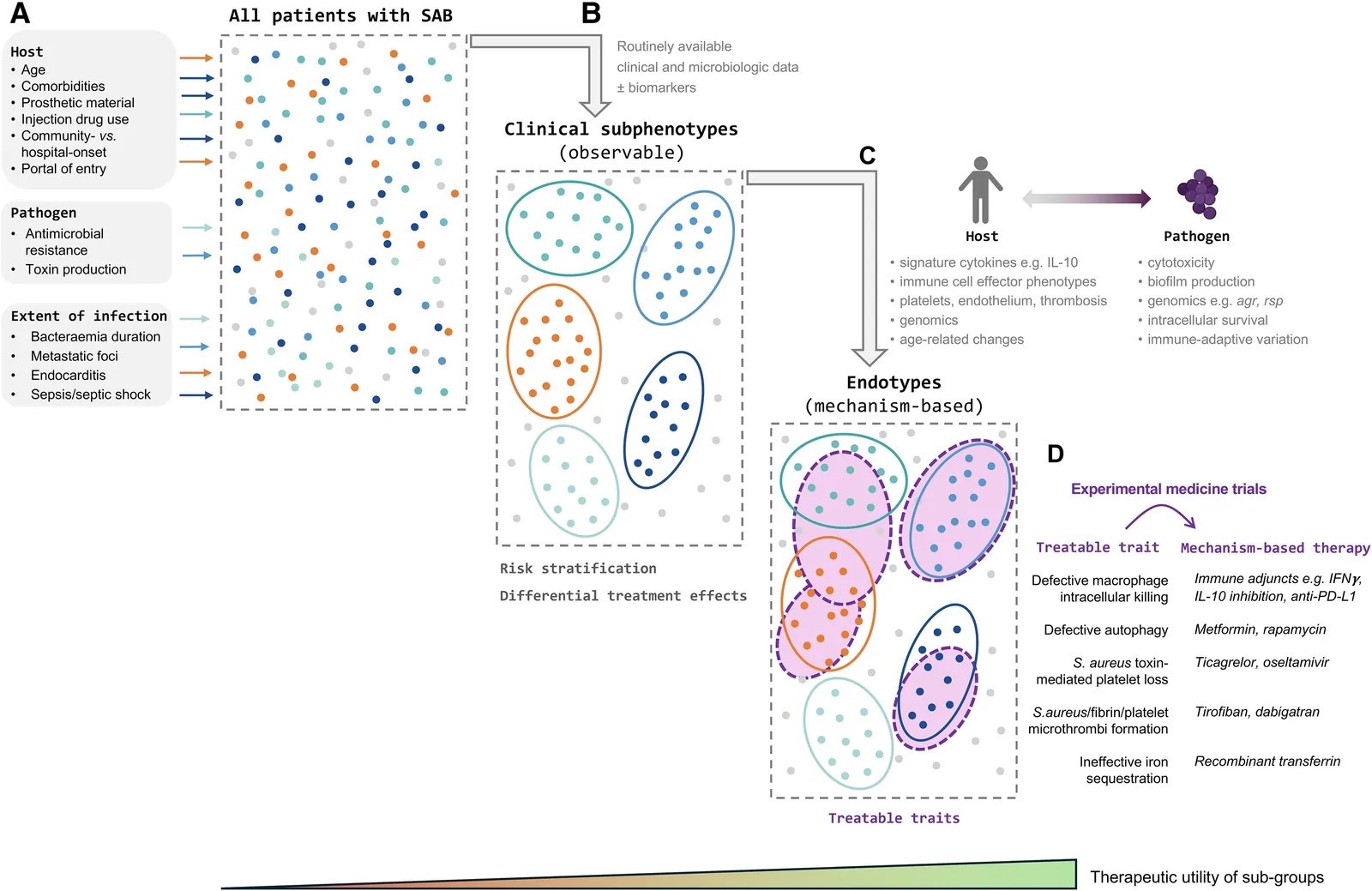
Current progress and future goals for addressing heterogeneity in SAB. *A*, SAB is intrinsically heterogeneous due to variation in host characteristics, the process underlying development of bacteremia, pathogen variation, and the extent of infection. Points represent individual patients, colors represent heterogeneity. *B*, Routinely available clinical data can be used to stratify patients into clinically informative subphenotypes, improving prognostication (*eg*, https://fen-aureus.com/) and potentially identifying subgroups of patients more likely to benefit or suffer harm from adjunctive rifampicin and fosfomycin. Groupings represent subphenotypes, with shared observable clinical characteristics. However, this approach does not address disease mechanisms. *C*, Integration of deep phenotyping of the host and pathogen has the potential to define mechanism-based SAB endotypes (purple groupings) and associated treatable traits, targetable using host-directed therapies. The extent to which mechanistic endotypes will overlap with clinical subphenotypes is currently unknown. *D*, Novel therapies can be investigated in precision experimental medicine trials, conducted in narrow populations enriched for patients hypothesized to benefit from the specific intervention being tested, based on treatable traits. IL-10: interleukin 10; PD-L1: programmed death-ligand 1; IFNγ: interferon gamma.

*Staphylococcus aureus* infections are a major cause of morbidity and mortality worldwide. The Global Burden of Disease Study identified *S. aureus* as the leading overall cause of death due to bacterial infection globally, and due to bacteremia specifically [1]. The clinical challenges posed by *S. aureus* bacteremia (SAB) remain as recognizable today as they did when first described more than half a century ago [2, 3]. A defining challenge is heterogeneity (Figure 1*A*), encompassing host characteristics (*eg*, age, sex [4], comorbidities), the process underlying development of bacteremia (*eg*, healthcare contact, portal of entry), infection manifestations (*eg*, metastatic foci, endocarditis, involvement of implanted prosthetic material), and bacterial characteristics (*eg*, toxin production, antimicrobial resistance, *spa* type/clonal complex [5, 6]). In addition to these observable features, interpatient differences in underlying molecular pathophysiology remain obscure. Clinical and biological heterogeneity in SAB pose significant challenges for the clinical management of individual patients and research efforts to develop improved treatment approaches. We contend that investigating SAB as a single disease is probably impeding the identification of beneficial therapeutic approaches.

## CURRENT DIFFICULTIES WITH PATIENT STRATIFICATION IN SAB

Currently, there is no consensus on reducing heterogeneity in clinical trials to achieve patient stratification. Amongst existing SAB trials, substantial intertrial variability exists in the characteristics of enrolled patients. This includes portal of entry, proportion with complicated SAB, patient comorbidities, and proportion of infections with methicillin-resistant *S. aureus* (MRSA) [7, 8]. Moreover, patients with SAB enrolled in trials differ significantly from nonstudy participants, including having a significantly lower risk of death [7]. This discrepancy is at least partially by design, as inclusion/exclusion criteria in trials frequently exclude patients not expected to survive the study period, to more accurately assess the efficacy of the intervention. As a result, it is possible that current trials fail to identify subgroups of patients who may differentially benefit or suffer harm from specific treatments. Definitions of “low risk” and “complicated” SAB have been proposed in response to this (Table 1) [9–12]. However, there are discrepancies in the overlap, agreement, and outcomes of existing “low risk” definitions when applied to the same patients (Supplementary Figure 1A,B,C) [13]. There is also limited overlap in the co-occurrence of the individual components of IDSA-defined “complicated” SAB in the same patients, suggesting patients meeting this definition remain heterogeneous (Supplementary Figure 1D,E) [14, 15].

**Table 1.** Current Definitions of “Low-risk” SAB

	SNAP EOS Day 7 [9]	Hendriks et al [10]	SABATO Trial [11]
Purpose of definition	Identifying patients for EOS on platform day 7	Stratifying requirement for routine imaging	Identifying patients for EOS
Components of definition			
*Portal of entry*	SSTI or IV catheter only	Not restricted (78% were IV catheter-related SAB)	Pneumonia excluded
*Prosthetic material*	Cardiac device, heart valve, vascular grafts in situ: excluded	Any in situ material: excluded	Orthopedic implant or cardiac device in situ: excluded (unless SSTI or IV portal of entry)
*Other*	–	Community-onset: excluded	Immunosuppression: excluded Liver disease or hemodialysis: excluded (unless SSTI or IV source) PWID: excluded
*Common components of all definitions*	Mandatory to meet definition: Negative follow-up blood cultures Afebrile at 72h No clinically apparent metastatic infection If IV catheter-related SAB: CVC/catheter removal		

SNAP: *Staphylococcus aureus* Network Adaptive Platform trial; EOS: early oral switch; SABATO: *Staphylococcus aureus* Bacteremia Antibiotic Treatment Options; SSTI: skin or soft tissue infection; IV: intravenous; PWID: person who injects drugs; CVC: central venous catheter.

There are several immediate clinical problems arising from this difficulty with patient stratification. First, the reproducible identification of patients who could be safely managed with an abbreviated duration of intravenous treatment remains problematic. For example, despite applying the same criteria, real world patients potentially eligible for inclusion in the SABATO trial were more likely to have SAB from an unknown source, thus potentially at higher risk of treatment failure [14]. Second, it is possible that subgroups of patients with a high risk of SAB-related complications and/or treatment failure (*eg*, left-sided endocarditis, deep-seated pyogenic metastatic foci) could benefit from combination antimicrobial therapy but remain undefined because of heterogeneity in current trial populations. Overall, there is an unmet need for patient stratification, to precisely define clinically relevant subgroups of patients for clinical practice and research.

## PROGRESS IDENTIFYING CLINICALLY RELEVANT SUBPHENOTYPES OF SAB

Recently, three data-driven approaches for patient stratification in SAB have been reported (Table 2; Figure 1*B*).

**Table 2.** Overview of Studies Describing SAB Subphenotypes

Study	Swets et al [16, 17]	FEN-AUREUS [18]	MRSA-GEIRAS-SEIMC [19]
Included cohorts	Edinburgh 1, *n* = 458 ARREST RCT, *n* = 758 [20] SAFO RCT, *n* = 214 [21] Edinburgh 2, *n* = 463 IDISA, *n* = 490 [22] SABG-PCS, *n* = 755 [23]	ISAC, *n* = 2128 [24] INSTINCT, *n* = 1217 [25] FEN-AUREUS, *n* = 1185 BACSARM RCT, *n* = 155 [26] SAFO RCT, *n* = 214 [21] Edinburgh, *n* = 915 [27]	*n* = 419
MSSA/MRSA	Both	Both	MRSA
Methodology	LCA	Two-step cluster analysis	PCA then LCA
Interpretation	**A.** SAB associated with older age and cardio-metabolic multimorbidity; **B.** nosocomial IV catheter-associated SAB; **C.** Community-onset metastatic SAB; **D.** SAB associated with CKD and hemodialysis; **E.** SAB associated with injection drug use.	Source phenotypes: **A.** Skin/soft tissue Subphenotype **1** Subphenotype **2** **B.** Vascular device Subphenotype **1** Subphenotype **2** **C.** Other/unknown Subphenotype **1** Subphenotype **2** Mortality-risk subphenotypes: **1.** Lower risk; **2.** Higher risk	**1.** Younger, community-onset, SSTI/unknown portal of entry; **2.** Older age, female, dementia, CKD, healthcare-associated; **3.** Nosocomial, IV catheter-related; **4.** Device/valve-associated, metastatic foci, endocarditis.
Outcomes	Mortality (highest A; lowest E) Microbiologic (worst C and E)	Mortality	Mortality (highest 2; lowest 1)
Differential treatment effects	Adjunctive rifampicin (ARREST RCT)	Adjunctive fosfomycin (BACSARM & SAFO RCTs)	…

ARREST: Adjunctive Rifampicin to Reduce Early mortality from *STaphylococcus aureus* bacteremia; RCT: randomized controlled trial; IDISA: Improved Diagnostic Strategies in *S. aureus* bacteremia; SABG-PCS: *S. aureus* Bacteremia Group Prospective Cohort Study; ISAC: International *Staphylococcus aureus* Collaboration; INSTINCT: INvasive *STaphylococcus aureus* INfections CohorT; LCA: latent class analysis; PCA: principal component analysis; IV: intravenous; CKD: chronic kidney disease; SSTI: skin or soft tissue infection.

### Swets et al

Six cohorts were analyzed (*n* = 3138 adults with SAB) from the United Kingdom, Spain, the Netherlands, and the United States [16, 17]. These cohorts encompassed a diverse range of *S. aureus* clonal complexes and a variable proportion of patients with MRSA infection (0–40%). Class defining variables (Supplementary Table 1) were chosen based on consensus, missingness in the derivation dataset, collinearity, and positivity, then clustered using latent class analysis (LCA). The number of classes was selected based on interpretability, size of the smallest class, and Bayesian information criterion (BIC) [28–30]. Five clinical subphenotypes were reproducibly identified: (A) SAB associated with older age and multimorbidity (cardiovascular disease and dementia), (B) nosocomial intravenous catheter-associated SAB in younger people with less comorbidities, (C) community-onset SAB, with an unknown portal of entry, metastatic foci, and high C-reactive protein, (D) SAB associated with chronic kidney disease, hemodialysis, and healthcare contact, and (E) SAB associated with younger age, acquisition through injection drug use, and metastatic foci. Eighty-four-day mortality was highest in subphenotype A and lowest in E. Persistent bacteremia or microbiologic failure were highest in subphenotypes C and E. Subphenotype B was associated with a low risk of mortality and of persistent bacteremia or microbiologic failure. In a post hoc analysis of the ARREST trial [20], adjunctive rifampicin increased 84-day mortality in subphenotype B and reduced microbiologic failure in subphenotype C.

### FEN-AUREUS

The FEN-AUREUS study used routinely available day zero/early clinical and laboratory variables (Supplementary Table 1) from multiple cohorts to assign adults with SAB into phenotypes and subphenotypes. In the derivation cohort (ISAC, *n* = 2128), an unsupervised two-stage clustering approach identified three phenotypes based on the probable portal of entry (A: skin/soft tissue; B: vascular device; C: other/unknown), with two mortality-risk subphenotypes within each (1: lower risk; 2: higher risk). Parsimonious probabilistic prediction models were subsequently derived to support patient-level classification, implemented as an open-access calculator (https://fen-aureus.com/). Application of these models in additional cohorts (INSTINCT, *n* = 1217; FEN-AUREUS, *n* = 1185; Edinburgh, *n* = 915) confirmed consistent prognostic separation [18, 27]. In the pooled BACSAFO post hoc analysis [31] of two trials of adjunctive fosfomycin (BACSARM [26] and SAFO [21]; *n* = 369; Table 2), risk subphenotypes were combined with IDSA complicated/uncomplicated criteria [12], creating four strata with a clear mortality gradient and differential responses to adjunctive fosfomycin. In the low-risk/uncomplicated stratum, outcomes were similar with monotherapy versus adjunctive fosfomycin. In contrast, in high-risk/complicated SAB, adjunctive fosfomycin increased treatment success and improved Desirability of Outcome Ranking (DOOR) profile.

### MRSA-GEIRAS-SEIMC

This multicenter, retrospective observational cohort study was conducted in 15 Spanish hospitals, exclusively studying adults with MRSA bacteremia (*n* = 419) [19]. First, principal component analysis was used to reduce the dimensionality of correlated variables. Secondly, LCA was performed to cluster patients into subphenotypes (Supplementary Table 1). Based on the lowest BIC and the highest entropy, a four-class model was selected. Four subphenotypes were thus identified: (1) younger patients with less comorbidity, an unknown or skin/soft tissue portal of entry, and community-onset; (2) older patients with increased comorbidity, and healthcare acquisition; (3) nosocomial SAB with an intravenous catheter source; and (4) SAB associated with metastatic foci, prosthetic heart valves, and infective endocarditis. Cox regression analysis showed that the risk of 90-day mortality was significantly higher in subphenotypes 2 and 4 compared to 1. There were no differences in the outcomes of the patients who received monotherapy compared to combination therapy across the subphenotypes, but this was not a trial dataset.

## COMPARISON OF CURRENT APPROACHES

**Figure 2.**
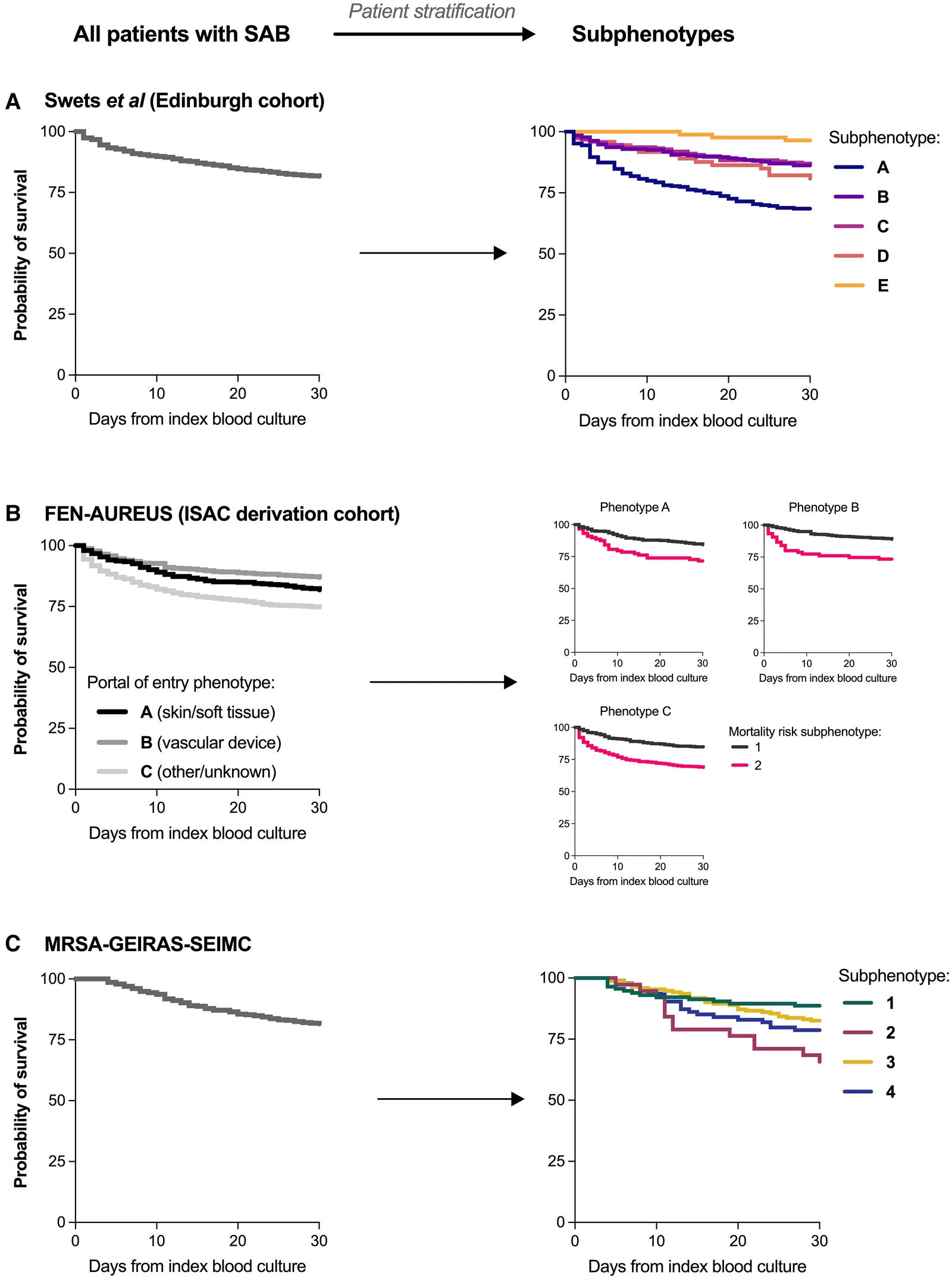
Identification of SAB subphenotypes with differences in risk of death. All-cause 30-d mortality, shown as Kaplan–Meier curves. Plots on left show mortality of entire patient cohort prior to stratification; plots on right show mortality stratified by subphenotype. *A*, Swets *et al* subphenotypes. Data shown Edinburgh 1 + 2 cohorts combined (*n* = 921) [16, 17]. *B*, FEN-AUREUS approach, deriving phenotypes based on portal of entry (left) then mortality risk subphenotypes (right). Data shown from the ISAC derivation cohort (*n* = 2128) [18]. *C*, MRSA-GEIRAS-SEIMC study subphenotypes (*n* = 419) [19].

Subgroups of patients with SAB can be identified using routinely available clinical variables and externally validated across independent datasets. These subgroups have clinically important differences in risk of death (Figure 2) and potentially differential treatment responses to adjunctive antimicrobial therapies. Age, co-morbidity, portal of entry, and acquisition were the predominant factors contributing to the clustering of patients into subgroups.

### Real-world Implementation

The FEN-AUREUS approach uses parameters available on day zero, reflecting baseline features and initial disease severity whereas the Swets et al and MRSA-GEIRAS-SEIMC approaches also utilize the presence/absence of metastatic infection. Metastatic foci are often identified many days after the index presentation. The FEN-AUREUS approach could therefore have immediate clinical utility for early risk stratification. Application of the Swets et al and MRSA-GEIRAS-SEIMC approaches require sequential reassessment and time for imaging to be completed.

### Included Patients

All current approaches are restricted to adults, highlighting the need to explore heterogeneity in children with SAB [32]. The Swets et al and FEN-AUREUS approaches were both robust in observational and trial cohorts, despite the recognized differences between these types of cohort [7, 8]. People who inject drugs were excluded from the SAFO trial and this variable was not included in the MRSA-GEIRAS-SEIMC clustering. In the Swets et al approach a distinct subphenotype associated with SAB acquired through injection drug use was reproducibly identified, indicating the relevance of this feature.

### Differential Treatment Effects of Adjunctive Antimicrobial Therapies

Taken together, findings from the Swets et al and FEN-AUREUS studies support a precision-based approach to combination therapy. In subgroups at lower risk of mortality and complications, adjunctive therapies offered no benefit. In fact, adjunctive rifampicin was associated with increased mortality in Swets et al subphenotype B, suggesting that risks of drug toxicity and drug-drug interactions outweigh potential benefits for patients at lower risk of SAB-related complications. In contrast, in carefully selected patients at higher risk of SAB-related complications, adjunctive agents could be beneficial, supporting further investigation of combination therapy confined to such subgroups. In BACSAFO, combining FEN-AUREUS risk subphenotypes with IDSA complicated SAB criteria identified an immediate antimicrobial stewardship message: avoid initiating combination therapy in uncomplicated SAB, while cautiously considering application and investigation in complicated phenotypes. The optimum duration of combination therapy remains unanswered and may differ between subgroups.

## LIMITATIONS OF CURRENT APPROACHES

### Mandatory Subgroup Membership

Interpretation, particularly of subphenotypes derived by LCA, is based on the “archetypal” patient (*ie,* an idealized description representative of the subphenotype overall [33]). However, all patients with available data are probabilistically assigned to a subphenotype, inevitably leading to intra-group variability and the associated risk of diluting important signals (*eg,* response to treatment). Ideally, an informative subphenotype would have a small amount of intra-group variability. To achieve this, a larger number of smaller but more homogeneous subphenotypes may be more informative, and some patients may be best not assigned to any.

### Dimensionality of Class Defining Variables

The current studies used routinely available clinical variables, chosen pragmatically to allow external validation across multiple cohorts. Inevitably, this has led to relatively low dimensionality of the class defining variables included. Potentially important omissions include more detailed physiologic data (*eg,* full SOFA score), biomarkers associated with SAB mortality and/or persistent bacteremia (*eg,* interleukin-1β, IL-10, IL-17A, CCL4 [34–39]), and routine microbiologic variables such as the number of positive blood cultures or time to blood culture positivity [40]. Included laboratory and physiologic variables were all from the time of the index blood culture. However, early changes (*eg,* in response to antimicrobial initiation) could also be informative as illustrated by the identification of trajectory-based sepsis subphenotypes using SOFA score changes during the first 72 hours of ICU admission [41].

### Differences in Mortality are not Necessarily Therapeutically Relevant

All-cause mortality has been used as the main outcome measure in the current studies. However, the extent to which differences in mortality are therapeutically relevant remains to be established. Death due to SAB can be the result of multiple pathways (*eg,* sepsis, heart failure in endocarditis, thrombosis, and decompensated chronic organ dysfunction) therefore may not differentiate patients based on disease mechanism. For example, two putative subgroups could have equivalent 30-day mortality, but in only one of them might *S. aureus*/fibrin/platelet interactions be causal in death and modifiable with dabigatran [42, 43]. Furthermore, although mortality remains the most important clinical outcome, it is not the only outcome that matters. A treatment strategy reducing the risk of treatment failure or relapse in patients not at high risk of death would also be desirable. Hierarchical composite endpoints such as DOOR address this, as well as increasing statistical efficiency, providing a useful addition to primary endpoints [44].

### Caution in Interpreting Post Hoc Differential Treatment Effects

The adjunctive rifampicin and fosfomycin findings arise from exploratory post hoc subgroup analyses with small sample sizes. While there is an overall consistency of direction across studies and drugs these observations are hypothesis-generating. Cautious interpretation and application are essential due to the frequent observation of toxicity associated with combination therapy in SAB.

## FUTURE GOALS

These approaches for identifying SAB subphenotypes represent an incremental improvement on the *status quo* but should be viewed as a starting point. We suggest there are four major goals for the future.

### Incorporate Pathogen Variation

Constrained by current clinical microbiology practices, current approaches lack inclusion of granular microbiologic data in clustering and therefore reflect host but not pathogen heterogeneity. However, within-species pathogen variation leads to important differences in pathogen-host interactions including escape from microbicidal responses [45], exemplified for *S. aureus* by the emerging MRSA lineage ST3390 [46]. In a study applying machine learning to predict SAB mortality (caused by CC22 and CC30), in vitro THP-1 (macrophage-like) cytotoxicity and biofilm formation were identified as predictors of mortality with a larger effect size than Charlson Comorbidity Index and age [47]. Bacterial genetic variants with smaller effect sizes were also identified from whole genome sequences. Interestingly these findings were specific to CC22. Considering persistent bacteremia, several in vitro pathogen traits have been associated with isolates from persistent versus resolving MRSA bacteremia: less hemolysis, less biofilm formation, more small colony variant formation at pH 4.0, and more intracellular small colony variant formation in immortalized dermal microvascular endothelial cells [48]. No differences in antimicrobial susceptibility between the isolates from persistent versus resolving bacteremia were identified. Naturally occurring loss of function mutations in the *rsp* transcription factor are associated with bacteremia (vs colonization) isolates. *In vitro* loss of *rsp* is associated with reduced hemolysis, upregulation of immunomodulatory factors, and reduced HeLa epithelial cell cytotoxicity, permitting improved intracellular survival [48, 49]. The extent to which *rsp* mutation varies between patients with different SAB subphenotypes or outcomes has not been systematically investigated. In addition, *agr* dysfunction has been linked to persistent SAB and higher mortality [50].

Together, these examples highlight the importance of incorporating relevant pathogen traits and genomics into patient phenotyping. In principle, this could include pathogen whole genome sequencing [51], virulence factor repertoires, and in vitro measurement of cytotoxicity, biofilm production, and intracellular persistence [52]. Current turnaround times of sequencing and in vitro assays, combined with a lack of standardized and quality-assured in vitro phenotyping assays, are barriers to implementation. However, whole genome sequencing turnaround times are decreasing, *eg,* a long-read pipeline including variant calling to identify transmission clusters has been described with a mean time from DNA extraction to useable analysis of 2 days [53]. Furthermore, it has recently been demonstrated that diagnostic MALDI-TOF spectra can quantify Agr-mediated cytotoxicity using the 3000 Da peak, which corresponds to the delta toxin [54]. Presence of the 3000 Da peak correlated with in vitro THP-1 cytotoxicity, and improved prediction of 30-day mortality compared to age and CCI alone. This metric is available at the time of SAB diagnosis if using MALDI-TOF.

### Actionable Risk Stratification in Clinical Practice

The subphenotypes discussed here can identify patient subgroups with an increased risk of death, and the FEN-AUREUS online calculator provides clinicians with a tool to do this on a ward round. Although improved prognostication may be helpful in routine clinical practice, we contend that a more ambitious goal is warranted: predicting *response* to specific therapeutic approaches, not simply *risk* of dying. Immediate applications of risk stratification using existing approaches and currently available therapies could be to reproducibly identify patients with favorable-prognosis subphenotypes as candidates for earlier intravenous-to-oral switch and/or abbreviated treatment courses. Swets et al subphenotype B (nosocomial intravenous catheter-associated SAB) represents a candidate subgroup for these approaches, with a low risk of death and microbiologic failure. Notably, intensified treatment with adjunctive rifampicin appeared harmful in this subphenotype. Conversely, subphenotypes associated with a higher risk of death (*eg*, FEN-AUREUS high risk subphenotypes) or microbiologic failure (*eg*, Swets et al subphenotype C, community-onset metastatic SAB) may warrant intensified treatment with combination therapy (fosfomycin in FEN-AUREUS and rifampicin in Swets et al).

### Narrow, Enriched Clinical Trials: Match the Intervention to the Patient

Investigating SAB as a single disease is likely to result in therapeutic options beneficial to hidden subgroups of patients being falsely rejected. SAB trials enroll heterogeneous populations with a variable proportion of patients at risk of SAB-related complications [7, 8]. This could explain why many trials have not detected benefits of combination therapy, whereas the Swets et al and FEN-AUREUS/BACSAFO approaches did in specific subphenotypes. Although these two examples of differential treatment effects come from post hoc stratification within trials, this is not what we consider to be the best application of patient stratification. Rather, the goal should be to prospectively match patients to potentially beneficial interventions, informed by post hoc differential treatment effects and, importantly, underlying pathophysiological differences. These narrow populations can then be enrolled in clinical trials, enriched for patients with a disease subphenotype hypothesized to benefit from the intervention being studied. This could be operationalized through platform trials with silos for different subphenotype-intervention combinations, modeled on the SNAP trial [55]. This concept is well exemplified by the design of the recent ImmunoSep trial in adults with sepsis, investigating precision immunotherapy [56]. Patients with a macrophage activation-like syndrome or sepsis-induced immunoparalysis were identified based on ferritin and CD45+/CD14+ monocyte HLA-DR expression, then randomized to either anakinra or interferon-gamma respectively. Against a backdrop of negative trials of immunotherapies in sepsis, this approach successfully identified treatment effects, providing proof of principle of the approach.

### Progress From Observable Phenotypes to Mechanistic Endotypes

Investigation of heterogeneity in immunobiology amongst patients with SAB is required to advance our understanding from clinically apparent subphenotypes toward mechanism-based disease endotypes, to deliver personalized therapies (Figure 1*C*). A systems-level understanding of host responses to *S. aureus* is emerging, identifying candidates for host-directed therapeutic targeting including *S. aureus*/fibrin/platelet interactions, phagocyte antimicrobial effector responses, iron homeostasis, autophagy, PD-L1 immune exhaustion, and metabolism [43, 57, 58]. For example, preventing *S. aureus* toxin-mediated platelet loss by re-purposing ticagrelor or oseltamivir improved outcomes in a mouse model of SAB [59] with a case report documenting possible benefit of ticagrelor in a patient with *S. aureus* endovascular infection and thrombocytopenia [60]. Interferon gamma can prevent serious infections in people with chronic granulomatous disease, where loss of phagocyte NADPH oxidase is associated with increased risk of invasive *S. aureus* disease [61]. A case report describes its use as an immune adjunct in persistent SAB, associated with increased leucocyte HLA-DR expression and clinical improvement [62]. Adjunctive interferon gamma has also shown potential efficacy in pulmonary non-tuberculous mycobacterial infection [63], and more recently (with or without anti IL-4/IL-13 dupilumab to reverse Th2 skewing) in disseminated coccidioidomycosis [64]. These provide proof of principle of benefit against pathogens with an intracellular lifestyle, which is increasingly recognized as a concept central to *S. aureus* pathogenesis [52, 65]. mTOR was prioritized by a meta-analysis of host responses to *S. aureus*, in association with other autophagy-related factors [43], potentially druggable by repurposing of metformin. Further investigation is supported by observational data identifying a reduction in mortality associated with metformin exposure amongst people with diabetes with SAB [66] and a role for autophagy in tolerance to *S. aureus* alpha toxin [67].

It is unlikely such host-directed approaches will benefit unselected patients with SAB equally. For example, there is a potential risk of inducing injurious hyper-inflammation by interferon gamma treatment in the absence of a defective antibacterial response [68]. In other cases, if the relevant pathophysiologic defect is not present, the intervention may simply not work (*eg,* thrombocytopenia, or *S. aureus*/fibrin/platelet microthrombus formation). Deep phenotyping will be required to identify biomarkers for therapeutically relevant differences in host responses between patients (treatable traits), allowing precision targeting of host-directed therapies in experimental medicine trials (Figure 1*D*). This concept is analogous to progress made in immune-mediated inflammatory disorders, where identification of “signature cytokines” enables precision targeting of immunomodulatory treatments [69]. Progress has even been made in the notoriously heterogeneous sepsis syndrome, with the reproducible identification of three consensus transcriptomic subtypes representing distinct pathophysiologic disturbances [70]. Early work in SAB has identified IL-10 as a potential signature cytokine. Higher IL-10 concentrations were associated with impaired T- and B-cell effector responses, differentiating persistent from resolving MRSA bacteremia [71]. Mechanistically, IL-10 can impair bacterial clearance indirectly by inhibiting T-cell Th1 polarization and interferon gamma production, and directly through inhibitory effects on macrophage intracellular bacterial killing [72, 73]. *Staphylococcus aureus*-induced IL-10 production also impairs T-cell responsiveness to experimental vaccines, and in mice IL-10 inhibition improved vaccine responsiveness [74]. Could a subgroup of patients with SAB and high circulating IL-10 benefit from IL-10 inhibition to recalibrate T-cell responses, or adjunctive interferon gamma to reconstitute defective macrophage intracellular killing? These responses are likely to be dynamic, potentially influenced by choice of antimicrobial therapy, so consideration of temporal trajectories will be informative [75]. Implementation of such approaches will require adoption of standardized quality-assured cytokine assays with turnaround times compatible with clinical decision making.

## CONCLUDING REMARKS

Major advances in the management of SAB will only be achieved by acknowledging and addressing the clinical and biological heterogeneity intrinsic to this infection, defining clinical subphenotypes and mechanism-based endotypes. This deep phenotyping will require systems-level multi-modal data and application of artificial intelligence such as machine learning. Understanding the therapeutic implications of this variability will allow targeted deployment of combination antimicrobial therapy, abbreviated treatment durations, and personalized host-directed therapeutic targeting. Heterogeneity should be rationalized to conduct clinical trials in narrow populations enriched for patients likely to benefit from the intervention being tested. SAB is not a single disease and trial design should reflect this with matching of investigational interventions to SAB subphenotypes or endotypes. Team science and data sharing to enable external validation of findings have been essential in generating the current evidence for patient stratification, and all authors of this review strongly advocate for expansion of open-source clinical science for SAB.

## Supplementary Material

jiag327_Supplementary_Data
