# Supplementary material for: Rationalizing Heterogeneity in Staphylococcus aureus Bacteremia: Current Progress and Future Goals
Source: J Infect Dis. 2026 Jun 30;234(3):e441–51. doi: 10.1093/infdis/jiag327 (PMC13600396; doi:10.1093/infdis/jiag327)
Supplement: Supplementary file 1 [file jiag327_supplementary_data.pdf]

Rationalising heterogeneity in *Staphylococcus aureus* bacteraemia: current progress and future goals.

SUPPLEMENTARY MATERIAL

Supplementary Table 1: Variables used for phenotype/subphenotype assignment in current studies

	**Swets *et al*[1, 2]**	**FEN-AUREUS[3, 4][b]**	**MRSA-GEIRAS-SEIMC[5]**
**Patient characteristics**	• Age<br>• Sex<br>• Dementia<br>• Vascular disease<br>• Cardiac prosthetic material<br>• Chronic kidney disease<br>• Liver disease<br>• Injection drug use	• Age<br>• Peripheral vascular disease<br>• Dementia<br>• Chronic kidney disease<br>• Congestive heart failure<br>• Any tumour<br>• Diabetes mellitus (± end-organ damage)<br>• Previous *S. aureus* infection in the last 12 weeks	• Age<br>• Sex<br>• Dementia<br>• Chronic kidney disease<br>• Liver disease<br>• Vascular disease<br>• Prosthetic heart valve
**Physiology[a]**	• Heart rate<br>• Temperature	• Oxygen saturation (ambient air)<br>• Glasgow Coma Scale	• SOFA score
**Laboratory results[a]**	• Haemoglobin<br>• Creatinine<br>• C-reactive protein	• Neutrophil count<br>• Creatinine	• Creatinine
**Microbiology**	• MRSA	• MRSA	–
**Clinical features of infection**	• Portal of entry<br>• Acquisition (Friedman's classification[6])	• Portal of entry<br>• Nosocomial acquisition<br>• Infection-related surgical wound<br>• Deep tissue infection or abscess at the time of index positive blood culture<br>• Pneumonia as the main focus of infection<br>• Main focus not established	• Portal of entry<br>• Acquisition (Friedman's classification[6])<br>• Presence of metastatic foci

[a]In all studies, patient physiologic and laboratory variables at the time of the index blood culture were used.
[b]In FEN-AUREUS, phenotypes were derived by unsupervised two-step clustering using 62 candidate variables. Portal of entry was the main feature separating the higher-order phenotypes. The variables listed here correspond to the parsimonious models used for patient-level subphenotype assignment in clinical practice rather than to the full set of variables entered into the original clustering procedure.

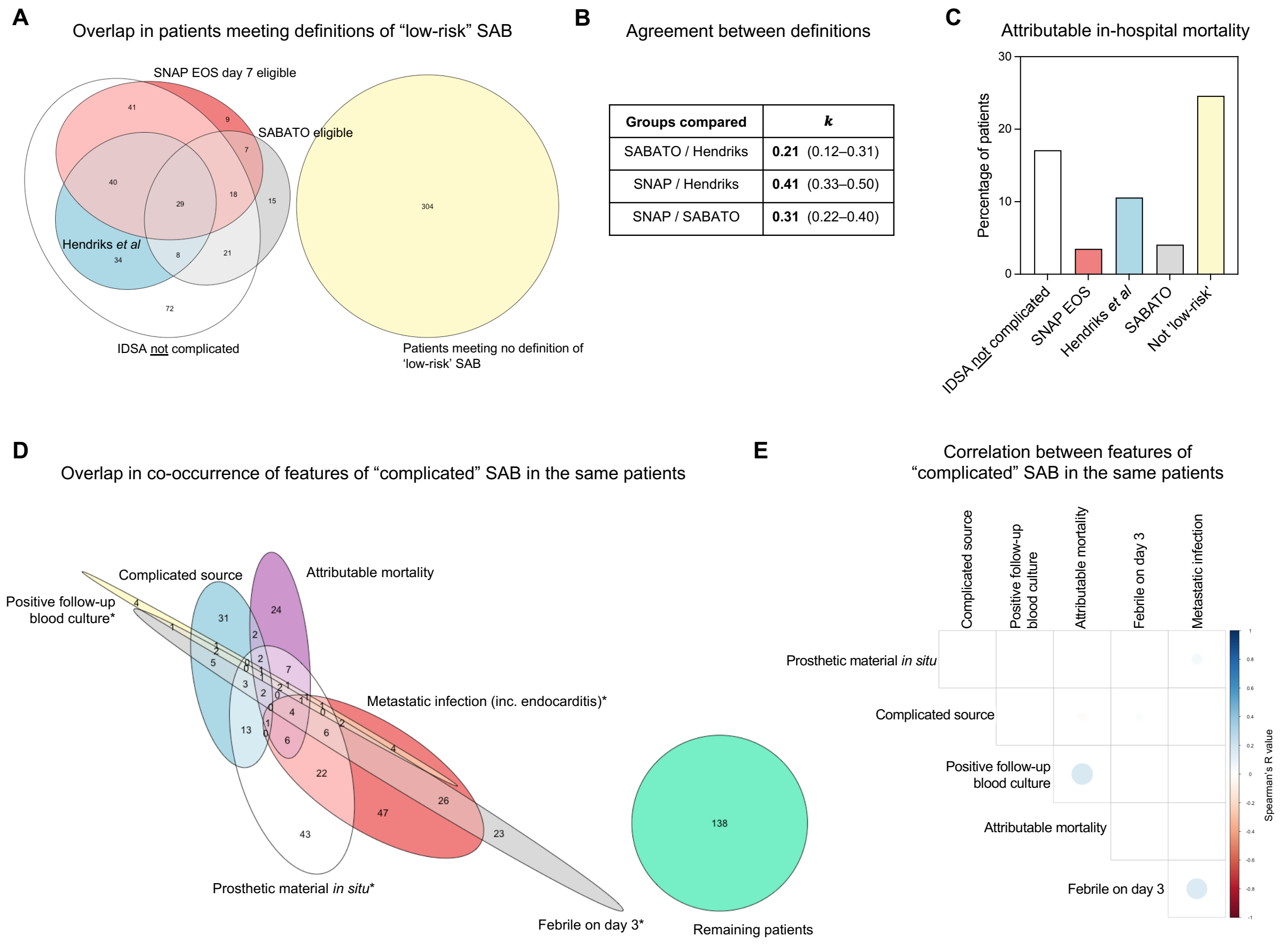

Groups compared	*k*
SABATO / Hendriks	**0.21** (0.12–0.31)
SNAP / Hendriks	**0.41** (0.33–0.50)
SNAP / SABATO	**0.31** (0.22–0.40)

Supplementary Figure 1: Application of current definitions of "low risk" and "complicated" SAB.

(A) Euler diagram illustrating the overlap in patients from the Edinburgh cohort (n=600) meeting current definitions of "low risk" SAB, modified from[7]. **(B)** Quantification of agreement between these definitions in the same patients using Cohen's kappa statistic, shown with 95% confidence intervals, modified from [7]. ***k*** ranges from 0 (no agreement) to 1.0 (close to perfect agreement). **(C)** Comparison of in-hospital attributable mortality between patients meeting the different definitions of "low risk" SAB. **(D)** Euler diagram illustrating the co-occurrence of features of IDSA "complicated" SAB in the same patients in the Edinburgh cohort, modified from[8]. Data for 461 patients with follow-up blood cultures collected. Variables marked with asterisk (*) are components of IDSA definition[9]. **(E)** Correlogram showing quantification of correlation between the features of IDSA "complicated" SAB in the same patients. Shaded cells indicate statistically significant Spearman correlations (*P* <0.05). Empty cells indicate no statistically significant correlation. Strength of blue shading represents a positive correlation.

SNAP: *Staphylococcus aureus* Network Adaptive Platform trial; EOS: early oral switch; SABATO: *Staphylococcus aureus* Bacteremia Antibiotic Treatment Options; IDSA: Infectious Diseases Society of America.

References

1. Swets MC, Bakk Z, Westgeest AC, et al. Clinical Subphenotypes of Staphylococcus aureus Bacteremia. Clin Infect Dis **2024**; 79:1153-61.

2. Swets MC, Bakk Z, Westgeest AC, et al. Reproducible identification of Staphylococcus aureus bacteremia clinical subphenotypes. Clin Infect Dis **2025**.

3. Escrihuela-Vidal F, Larrauri JG, López-Contreras J, et al. Subphenotype- and complication-guided adjunctive fosfomycin versus standard monotherapy in Staphylococcus aureus bacteraemia: pooled analysis of two randomised trials. The Lancet Regional Health – Europe.

4. Gutiérrez-Gutiérrez B, Gallego-Mesa B, Kaasch AJ, et al. Identification and validation of clinical phenotypes in Staphylococcus aureus bloodstream infection and their association with mortality (FEN-AUREUS study). EClinicalMedicine **2025**; 83:103240.

5. De La Villa S, Fernández-Hidalgo N, Escrihuela-Vidal F, et al. Analysis of Clinical Subphenotypes in Methicillin-Resistant Staphylococcus aureus Bacteremia: A Post Hoc Analysis of the MRSA-GEIRAS-SEIMC Study. Open Forum Infectious Diseases **2025**; 12.

6. Friedman ND, Kaye KS, Stout JE, et al. Health care--associated bloodstream infections in adults: a reason to change the accepted definition of community-acquired infections. Ann Intern Med **2002**; 137:791-7.

7. Berry K, Dewar S, Dolby HW, Cooper G, Sutherland RK, Russell CD. O03 Are proposed definitions of low-risk Staphylococcus aureus bacteraemia (SAB) clinically useful? JAC-Antimicrobial Resistance **2025**; 7.

8. Russell CD, Berry K, Cooper G, et al. Distinct Clinical Endpoints of Staphylococcus aureus Bacteraemia Complicate Assessment of Outcome. Clin Infect Dis **2024**; 79:604-11.

9. Liu C, Bayer A, Cosgrove SE, et al. Clinical practice guidelines by the infectious diseases society of america for the treatment of methicillin-resistant Staphylococcus aureus infections in adults and children: executive summary. Clin Infect Dis **2011**; 52:285-92.
